# Contralateral Cutaneous T-cell Lymphoma as a Mimicker of Multi-decade Atopic Dermatitis

**DOI:** 10.7759/cureus.114292

**Published:** 2026-08-10

**Authors:** Acacia Dasher, Catherine Tawfik, Katerina Warda, Aurel Apple, Ronald Brancaccio, Cindy Hoffman, Charles Gropper

**Affiliations:** 1 Dermatology, Pacific Northwest University of Health Sciences, Yakima, USA; 2 Dermatology, St. Barnabas Hospital Health System, New York, USA; 3 Dermatology, New York University, New York , USA

**Keywords:** ctcl: cutaneous t-cell lymphoma, ctcl mimicking atopic dermatitis, cytotoxic t lymphocyte (ctl), diagnostic delay, epidermotropism, indolent cutaneous lymphoma, lymphocytic exocytosis, mycosis fungiodes

## Abstract

Cutaneous T-cell lymphoma (CTCL) encompasses a group of low-grade, non-Hodgkin lymphomas that pose significant diagnostic challenges owing to variable clinical presentations and an indolent natural history. The disease is well established as a mimicker of benign inflammatory dermatoses, including atopic dermatitis (AD) and psoriasis, and diagnosis is further complicated by transient clinical improvement with topical corticosteroids, which may concurrently obscure histopathological findings on biopsy.

We report the case of a 75-year-old male with a 25-to-30-year history of treatment-refractory pruritic eruptions confined to a single palm and single foot, initially attributed to AD. Despite multiple prior nondiagnostic skin biopsies, a repeat broad shave biopsy of the right medial Achilles region performed five months after an initial nonconclusive punch biopsy of the left hypothenar eminence revealed epidermotropic CD4+ T-cell infiltrates with significant loss of CD7 expression and a CD4:CD8 ratio of 5:1, establishing a diagnosis of CTCL consistent with mycosis fungoides (MF). The patient was subsequently referred to a tertiary oncology center for formal disease staging and management. This case underscores the importance of maintaining a high index of suspicion for CTCL in patients with chronic, recalcitrant dermatitis, the critical value of topical steroid cessation prior to tissue sampling, and the utility of repeat biopsy with comprehensive immunohistochemical profiling in arriving at a timely and accurate diagnosis.

## Introduction

Cutaneous T-cell lymphoma (CTCL) encompasses a group of malignant, low-grade, non-Hodgkin lymphomas characterized by variable presentations and an indolent natural history, making accurate and timely diagnosis a significant clinical challenge [1,2]. Clinically, CTCL can manifest as patches, plaques, and tumors and is well recognized as a mimicker of benign dermatologic conditions such as eczematous dermatitis and psoriasis [2,3]. Patients often describe pruritic, persistent lesions that recur over several years, further obscuring the underlying diagnosis [3]. The most common presentation involves progressive pruritic lesions arising on sun-protected areas such as the trunk, with cutaneous findings ranging from patches to plaques and tumors; additional features may include alopecia, lymphadenopathy, and thickening of the palms and soles (palmoplantar keratoderma) [4,5]. The insidious onset of disease, combined with its propensity to simulate benign inflammatory processes, frequently results in significant diagnostic delay [2,3]. This challenge may be further compounded by the transient clinical improvement conferred by long-term topical corticosteroid use, which can temporarily mask the true underlying pathology [2,6]. Of particular importance, the continued use of topical steroids proximate to the time of biopsy has been shown to alter histopathological findings, potentially obscuring the characteristic lymphocytic infiltrates necessary for a definitive diagnosis and further contributing to diagnostic delay [2,6].

Here we present a unique case of a 75-year-old man with a 30-year history of cutaneous findings on a single palm and single foot, initially attributed to eczematous dermatitis refractory to topical steroids, and ultimately diagnosed as CTCL. Notably, our patient demonstrated no cutaneous involvement of the trunk or other typical sites, representing an atypical and diagnostically elusive presentation. After multiple nonconfirmatory skin biopsies, immunohistochemistry revealed CD3+ T-cells with co-expression of CD5 and focal loss of CD7, confirming the diagnosis. This case serves as an important reminder that maintaining a high index of suspicion for CTCL in the setting of chronic, treatment-resistant dermatoses, alongside ensuring proper cessation of topical steroids prior to tissue sampling, are critical steps in facilitating accurate histopathological interpretation, timely diagnosis, and ultimately, improved patient outcomes.

## Case presentation

A 75-year-old male with a past medical history significant for atopic dermatitis (AD) presented to the outpatient dermatology department with a pruritic rash on the left hand and right foot, present for the past 25 to 30 years. He described the rash as itchy, flaky, and painful, with a prior erratic and poor response to topical steroid treatments. On physical examination, erythematous atrophied patches with honey-colored crusting were distributed on the left hand and right foot. Additional findings of erythematous scaly plaques with maceration in a moccasin-like distribution were discovered on the right foot (Figure 1). He had previously failed therapies, including prolonged durations of potent topical corticosteroids, including clobetasol propionate cream 0.05% and halobetasol propionate ointment 0.05%, as well as antifungal therapy with ketoconazole 2% cream. Due to skin atrophy from prolonged steroid use, topical ruxolitinib cream 1.5% was initiated and resulted in a small improvement. Subsequently, the patient was started on standard dosing of dupilumab injections and mupirocin ointment as needed for impetiginized areas.

**Figure 1:**
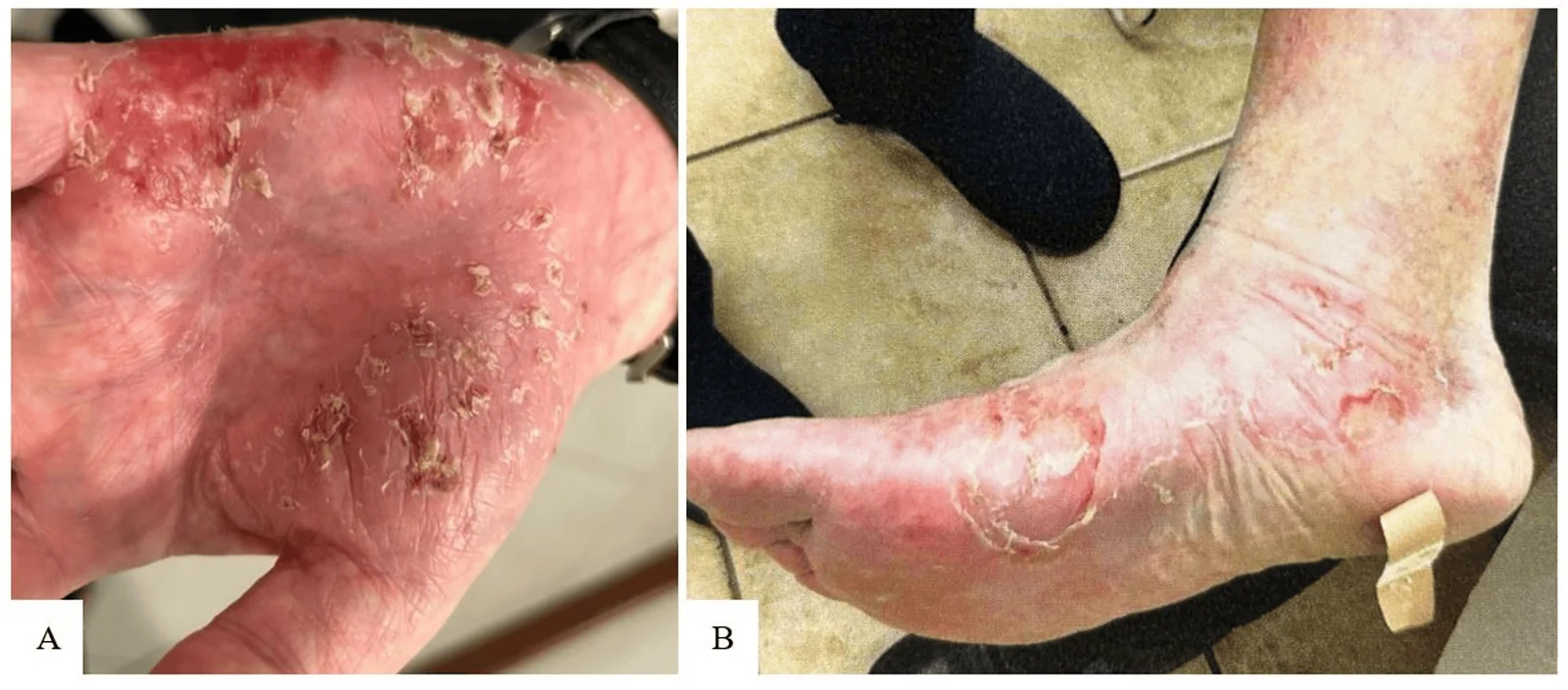
Rash on unilateral palm and foot A: Left hypothenar eminence exhibiting atrophic, yellow-crusted, erythematous, and scaly patches and plaques; B: Right medial Achilles skin exhibiting a scaly, yellow-crusted patch

The patient responded well initially to dupilumab therapy, but after three months, recurrent flare-ups prompted further investigation via a 3 mm punch biopsy of the left hypothenar eminence, which exhibited a macerated, atrophic, crusted, erythematous, and scaly patch. Histopathology demonstrated a superficial perivascular infiltrate with numerous lymphocytes extending into the overlying epidermis with a relative lack of spongiosis. Many of the lymphocytes exhibited small nuclei with regular nuclear contours. The epidermis was irregularly hyperplastic with compact orthokeratosis. A periodic acid-Schiff with diastase (PAS-D) stain failed to reveal fungal hyphae. Immunoperoxidase studies revealed the infiltrate to be comprised of CD3+ T-cells with co-expression of CD5 and only focal loss of CD7 expression (Figure 2). The ratio of CD4+ to CD8+ lymphocytes was 3:1. There was only focal reactivity for PD1. The proliferation fraction for Ki67 was low. A small subset of lymphocytes reacted for CD20, and CD30 was negative. The microscopic diagnosis was determined to be atypical lymphoid infiltrate with prominent lymphocytic exocytosis, with findings most likely consistent with a reactive nature. The patient continued his current treatment regimen; however, his rash continued to be inadequately controlled, prompting an additional biopsy five months later.

**Figure 2:**
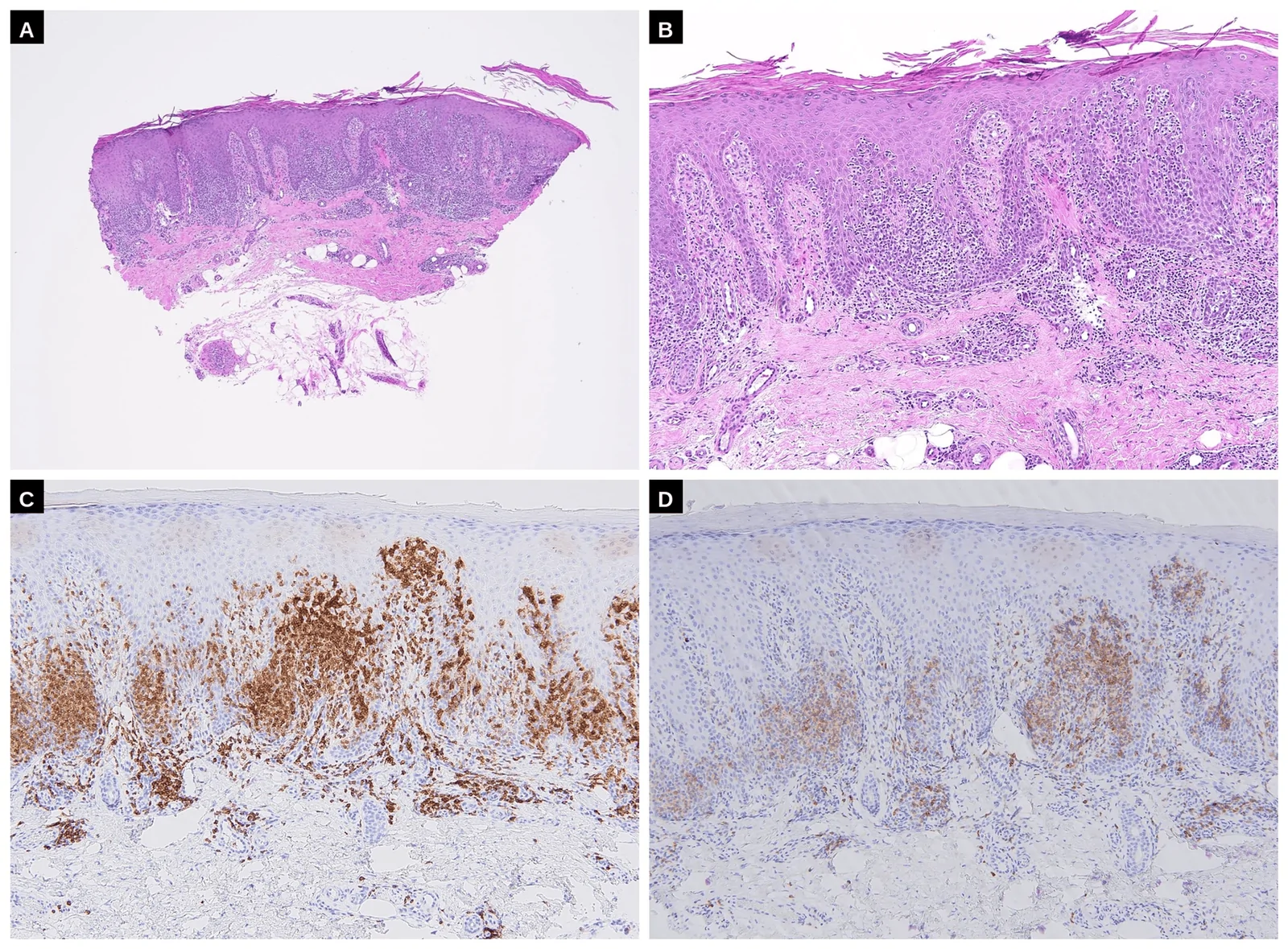
Histopathology of the first biopsy (left hypothenar eminence, 3 mm punch, accession 44112) A: Low-power view (H&E, 4×) demonstrating compact orthokeratosis, irregular epidermal hyperplasia, and superficial perivascular lymphocytic infiltrate with epidermotropism. B: High-power view (H&E, 10×) showing lymphocytic exocytosis with small lymphocytes, regular nuclear contours, and relative lack of spongiosis, consistent with a reactive process. C: CD3 immunostain (10×) confirming predominantly T-cell composition of the infiltrate. D: CD7 immunostain (10×) demonstrating only focal CD7 loss with partial preservation of staining within intraepidermal lymphocytes.

A second biopsy via the broad shave method was performed on a thin, scaly, macerated patch at an alternate site on the right medial Achilles. Histopathology demonstrated a superficial perivascular lymphocytic infiltrate. Lymphocytes extended into the overlying epidermis, where there was a relative lack of spongiosis. Within the epidermis, many of the lymphocytes exhibited enlarged hyperchromatic nuclei with irregular nuclear contours. Immunoperoxidase studies revealed the epidermotropic lymphocytes to be comprised predominantly of CD4+ T-cells with preservation of CD3 and CD5 expression but with significant loss of CD7 expression (Figure 3). The ratio of CD4+ to CD8+ lymphocytes was 5:1. CD30 highlighted only a small subset of lymphocytes, and CD20 was negative. The microscopic diagnosis was determined to be cutaneous T-cell lymphoma (CTCL). Given the biopsy-confirmed diagnosis, the patient was referred to a tertiary oncology department for comprehensive laboratory staging, including complete blood count with differential, lactate dehydrogenase, and peripheral blood flow cytometry, as well as formal disease staging and treatment planning. The patient is pending follow-up with dermatology.

**Figure 3:**
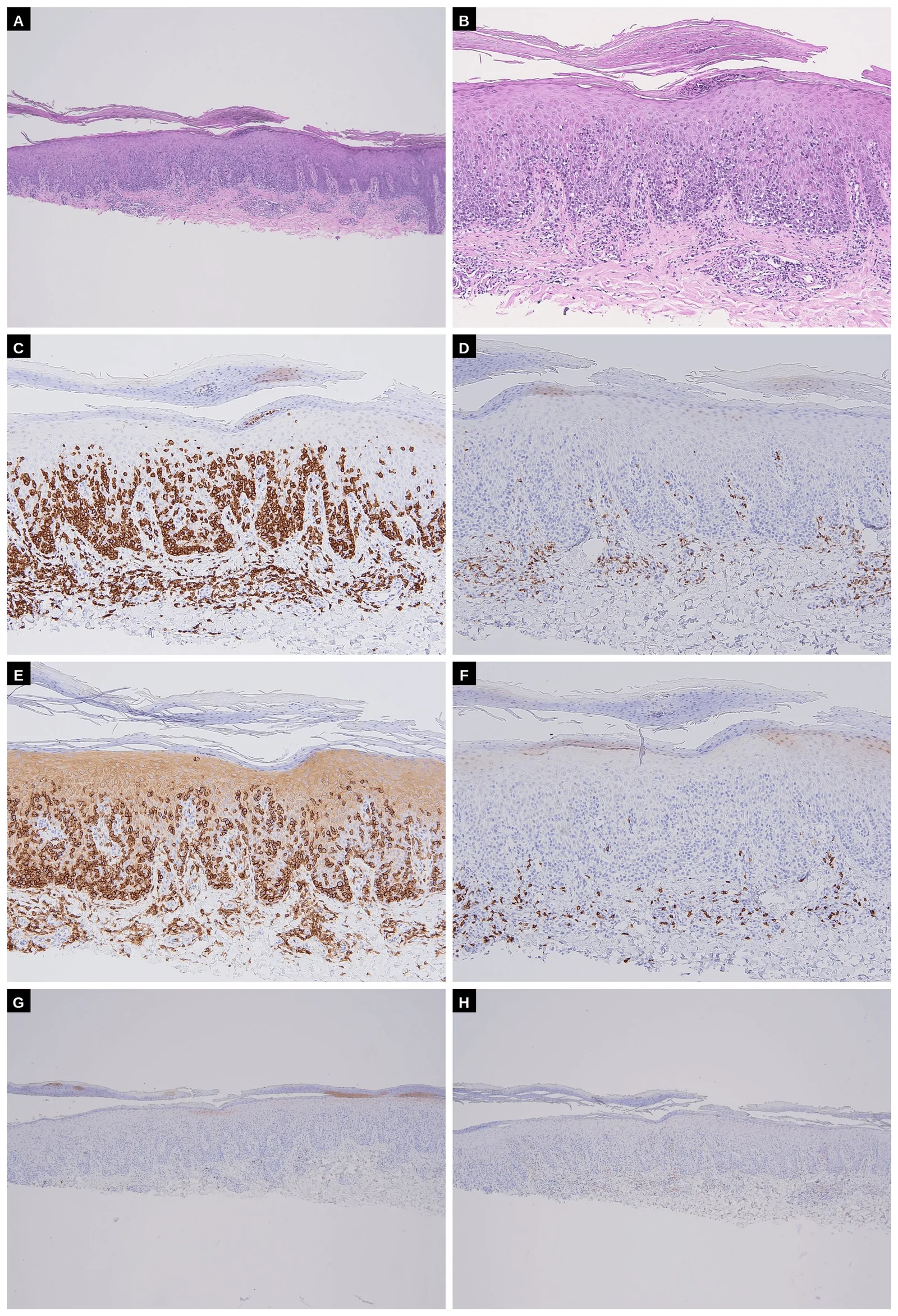
Histopathology of the second biopsy (right medial Achilles, broad shave, accession 77625) A: Low-power view (H&E, 4×) showing compact orthokeratosis, irregular epidermal hyperplasia, and dense epidermotropic lymphocytic infiltrate. B: High-power view (H&E, 10×) demonstrating epidermotropic lymphocytes with enlarged hyperchromatic nuclei and irregular nuclear contours, with relative lack of spongiosis, consistent with cutaneous T-cell lymphoma. C: CD3 immunostain (10×) confirming preserved T-cell marker expression. D: CD7 immunostain (10×) showing marked loss of CD7 in the epidermotropic population, with CD7+ cells largely restricted to the dermis — a key distinguishing feature from the first biopsy (Figure 2D). E: CD4 immunostain (10×) demonstrating a predominant CD4+ epidermotropic population. F: CD8 immunostain (10×) highlighting the minority CD8+ population, consistent with a CD4:CD8 ratio of 5:1. G: CD20 immunostain (4×) showing negative staining, excluding a B-cell lymphoproliferative process. H: CD30 immunostain (4×) with only focal positivity in a small subset of lymphocytes, consistent with mycosis fungoides rather than a CD30+ cutaneous T-cell lymphoproliferative disorder.

## Discussion

The indolent nature and variability of CTCL can make diagnosis difficult, usually requiring multiple biopsies to confirm the etiology [2,3]. The benign and malignant stages of CTCL can be difficult to distinguish due to similar histologic and immunologic findings in circulating T-cells, with only slight differences in surrogate cell-surface markers [3]. The relationship between CTCL and benign cutaneous conditions is not well understood, in part because CTCL mimics other disease processes and undergoes a slow transition between benign-appearing and overtly malignant states [2,3]. Additionally, CTCL can initially improve with topical steroid treatment, delaying diagnosis and treatment [2]. Topical steroid use has also been associated with an increase in inconclusive histopathological results [2]. Cutaneous T-cell lymphoma is a relatively rare malignancy with an incidence of 8.55 per million people, a median age of onset of 55 years, and a 2:1 male predominance [4,7].

Cutaneous T-cell lymphoma can be differentiated from other cutaneous lymphomas via immunohistochemistry (IHC). The role of CD markers has allowed for differentiation between B-cell and T-cell lymphomas, with T-cell lymphomas typically demonstrating CD3+, CD20-, and CD45+ staining, and B-cell lymphomas typically presenting with CD3-, CD20+, and CD45+ staining [1]. The most common primary cutaneous T-cell lymphoma is mycosis fungoides (MF), which typically demonstrates a CD4-predominant epidermotropic infiltrate with a CD4:CD8 ratio greater than 3:1, preservation of CD3 and CD5 expression, and variable loss of CD7 [1,5,7]. The CD30 expression serves as a key marker distinguishing MF from CD30+ cutaneous T-cell lymphomas such as lymphomatoid papulosis and primary cutaneous anaplastic large cell lymphoma; a CD30-negative to focally positive phenotype, as observed in our patient, is consistent with MF [1,6]. Classic MF can be differentiated from the Sézary syndrome variant of CTCL by the CD4:CD8 ratio, which is typically elevated (>3:1) in classical MF and markedly elevated (>10:1) in Sézary syndrome [8].

Our patient's extensive history of recalcitrant AD and subsequent histologic findings are consistent with CTCL, although his clinical presentation differed from the typical distribution seen in this disease. Our patient represents a unique case of CTCL with lesions confined to the unilateral palm and foot, which are sun-exposed areas, rather than the classic presentation on the sun-protected trunk. The patient's cutaneous findings created a diagnostic dilemma as a mimicker of AD, given the initial improvement of lesions with topical steroids followed by repeated recurrences over 25 to 30 years, with partial responses also seen to therapies used in the management of AD, including topical ruxolitinib cream and dupilumab injections. Additionally, the patient's atrophic scaly plaques were initially attributed to topical steroid overuse; however, there is a high likelihood they represented the atrophic variant of CTCL. The patient's second biopsy demonstrated IHC findings consistent with MF, including CD3+, CD20-, and CD45+ staining and a CD4:CD8 ratio of 5:1. The first biopsy result may have been confounded by concurrent topical steroid use, resulting in nonspecific findings of lymphocytic exocytosis, which can be associated with reactivity and chronic inflammation [6].

There is no cure for CTCL; therefore, treatment focuses on improvement or complete clearance of lesions with the goal of achieving remission. The course of treatment depends on the stage of disease. Early stages (IA and IB) utilize skin-directed therapies such as high-potency corticosteroids, ultraviolet phototherapy, and sometimes radiotherapy. For intermediate stages (IIa to IIIb), systemic therapies such as low-dose interferon-alfa and extracorporeal photopheresis have demonstrated efficacy. Late-stage disease (stage IVa1 and beyond) is primarily managed with chemotherapeutic regimens, which are considered palliative given a pattern of robust initial response followed by eventual disease progression [6]. A limitation of this report is that formal laboratory staging data, including peripheral blood studies and complete blood count with differential, were not available from the dermatology encounter, as these investigations were deferred to the oncology team upon biopsy confirmation of CTCL.

## Conclusions

This report highlights the variability in presentation and quiescent nature of CTCL, which can lead to inconclusive IHC results and a subsequent delay in diagnosis. Our case demonstrates the importance of repeat biopsy in the setting of poor treatment response or prior histologic reports demonstrating lymphocytic exocytosis. The initial inconclusive results from the first biopsy further underscore the importance of discontinuing topical steroid use at least two weeks prior to tissue sampling from any suspected inflammatory dermatitis, given the risk of false-negative identification of CTCL. Our case elucidates a prolonged course of cutaneous findings on the palm and foot spanning over three decades, with lesions masquerading as eczematous dermatitis. Notably, unilateral palmoplantar involvement, while a recognized but exceptionally rare manifestation of CTCL, further underscores the diagnostic complexity of this case; clinicians should maintain a high index of suspicion for cutaneous lymphoma even when distribution patterns deviate from classic presentations. This further illustrates that atypical localization, in concert with histological suppression from prior corticosteroid use, can substantially delay accurate diagnosis. Although CTCL can be diagnostically challenging, timely intervention is imperative for effective management and improved patient outcomes. Our findings support the pivotal role of IHC in confirming the diagnosis and guiding treatment and staging decisions in CTCL.
